# Parallel Algorithm on GPU for Wireless Sensor Data Acquisition Using a Team of Unmanned Aerial Vehicles

**DOI:** 10.3390/s21206851

**Published:** 2021-10-15

**Authors:** Vincent Roberge, Mohammed Tarbouchi

**Affiliations:** Department of Electrical and Computer Engineering, Royal Military College of Canada, Kingston, ON K7K 7B4, Canada; tarbouchi-m@rmc.ca

**Keywords:** data acquisition, genetic algorithm, graphics processing units, path planning, parallel computing, unmanned aerial vehicle, wireless sensors

## Abstract

This paper proposes a framework for the wireless sensor data acquisition using a team of Unmanned Aerial Vehicles (UAVs). Scattered over a terrain, the sensors detect information about their surroundings and can transmit this information wirelessly over a short range. With no access to a terrestrial or satellite communication network to relay the information to, UAVs are used to visit the sensors and collect the data. The proposed framework uses an iterative k-means algorithm to group the sensors into clusters and to identify Download Points (DPs) where the UAVs hover to download the data. A Single-Source–Shortest-Path algorithm (SSSP) is used to compute optimal paths between every pair of DPs with a constraint to reduce the number of turns. A genetic algorithm supplemented with a 2-opt local search heuristic is used to solve the multi-travelling salesperson problem and to find optimized tours for each UAVs. Finally, a collision avoidance strategy is implemented to guarantee collision-free trajectories. Concerned with the overall runtime of the framework, the SSSP algorithm is implemented in parallel on a graphics processing unit. The proposed framework is tested in simulation using three UAVs and realistic 3D maps with up to 100 sensors and runs in just 20.7 s, a 33.3× speed-up compared to a sequential execution on CPU. The results show that the proposed method is efficient at calculating optimized trajectories for the UAVs for data acquisition from wireless sensors. The results also show the significant advantage of the parallel implementation on GPU.

## 1. Introduction

Wireless Sensor Networks (WSNs) are composed of a large number of sensor nodes deployed to monitor physical phenomena. Coupled with the expansion of the Internet of Things, WSNs enable new applications such as intelligent transportation, water distribution and collection networks and the smart grid. WSN are also used in home and industry automation, plant monitoring, scientific experiments, forest monitoring, border patrolling, environmental observation, machine and structure health monitoring, security enhancement, underwater world observation, water quality monitoring, air pollution examination, landslide detection, and natural disaster prevention [1]. In a military context, WSNs can be used to monitor a terrain for ground troop or vehicle movements. In these applications, sensor nodes are low-power devices often using the IEEE 802.11af and 802.15 protocols and capable of communicating over a short range [2]. In typical WSNs, sinks or gateways are installed to collect the information from the wireless sensor nodes and to forward the data to the internet. However, in the case of a remote WSNs such as in military theater or in emergency situations where the WSNs are rapidly deployed in the environment, there is usually no infrastructure in place to accept the gateways. In such situation, UAVs have been proposed as an alternative to static gateways [3,4,5]. The UAVs act as mobile sinks and visit each sensor node, download their data and relay it back to a base station. Compared to unmanned ground vehicles, UAVs can cover more terrain in a shorter period of time. They can also be used in a rugged environment that would be difficult or impossible to reach by land. This is especially the case for landslide and avalanche prediction. With UAVs, it is also possible to ensure line of sight (LOS) with the wireless sensors by controlling the position of the UAVs. To accelerate the data collection, a team of collaborating UAVs can also be used.

The use of UAVs to assist terrestrial communication networks is quite recent, but has seen a rapid increase of interest by researchers in recent years. In their survey paper, Alzahrani et al. [6] provide the state-of-the-art in applications and challenges for UAV assisted networks. They highlight the benefits of the UAV assistance paradigm, such as providing a cost-effective alternative for ground networks’ limitations, a dependable coordination with ground network infrastructure, an efficient eye in the sky of the area covered, a robust deployable backup network for damaged terrestrial network infrastructures, and a communication bridge between existing networks and isolated users. However, their study also mentions the numerous challenges and technical issues related to the deployment of UAVs for the assistance of terrestrial networks that remains to be investigated and solved. Some of the challenges identified by the authors of [6] are UAV coordination, routing of messages, data gathering or data collection, UAV monitoring, communication with cellular networks, communication with Internet of Things networks, use of UAVs for disaster management, distributed computing, and secure communication. Another noteworthy survey paper on UAV assistance to terrestrial networks was published by Oubbati et al. [7]. This paper focuses on the networking scheme used by the UAVs and identifies Software-Defined Network and Network Function Virtualization as two promising technologies to support UAV assistance to terrestrial networks. They discuss several use cases and challenges related to UAV-assisted network and identify future research directions with a focus on the softwarization of UAV networks.

This present paper addresses the problem of UAV assistance to terrestrial communication networks by focusing on the challenge of data collection using UAVs. In the absence of a reliable communication network to relay the data from the wireless sensors to a monitoring station, UAVs are used to visit the wireless sensors, download their data, and relay the data to the base station. This problem is known as the data mule problem [8]. A concrete example of the data mule problem would be in a military operation where wireless sensors are deployed from an aircraft over a terrain to monitor the environment and UAVs are used to collect the information from the sensors. Following a natural disaster such as flooding, sensors could also be deployed to monitor the water level and UAVs could be used to collect the information from the sensors. There are many other scenarios where UAVs would be helpful to collect the data from wireless sensors. In this UAV-based data collection, the path planning and coordination of the UAVs is a complex problem, especially if done in a complex 3D environment with obstacles.

This present paper proposes a framework for the wireless sensor data acquisition using a team of UAVs. The sensors are scattered over a 3D environment which also includes some no-fly zones or obstacles that must be avoided by the UAVs. The proposed framework uses an iterative method based on the k-means clustering algorithm to group the sensors into clusters and identify the location of DPs, locations where the UAVs hover to download the data from the sensors that are part of the cluster. A sensor is associated to a DP when it is within communication range and in line of sight. After the locations of the DPs have been calculated, the framework uses a SSSP algorithm to find the optimal path between all possible pairs of DPs. This ensures that the final trajectory for each UAV includes the sequence of DPs to visit, but also the path between the DPs. The SSSP algorithm uses multiple grids of nodes superposed at the same location to impose a cost when changing direction. After the SSSP has run, a Genetic Algorithm (GA) is used to assign the DPs to the UAV and to find the order in which the DPs are visited. The GA is supplemented with a 2-opt local search heuristic to accelerate convergence. The results of the SSSP and the GA are combined to build complete trajectories for each UAV. A model of the UAV based on experimental flights is used to estimate the speed and the power consumption of the UAVs along their trajectory. Finally, a collision-avoidance strategy is implemented to ensure collision-free trajectories. Being the most time-consuming part of the framework, the SSSP algorithm is parallelized on a Graphics Processing Unit (GPU) and executes 33.3× faster than the sequential implementation on CPU. The framework is tested on two different 3D maps with scenarios containing 70 and 100 sensors grouped into 15, 40, and 55 DPs.

This paper makes three main contributions which highlight its novelty compared to previous works. Firstly, it presents a complete framework for the problem of wireless sensor data acquisition using multiple UAVs including optimized trajectories between DPs and a collision-avoidance strategy. Secondly, it expands on previous research by solving the problem in a 3D environment instead of 2D. Thirdly, it parallelizes the SSSP algorithm on GPU to reduce the runtime of the overall framework by a factor of 33.3× which allows for the fast computation of the UAV trajectories.

The rest of the paper is organized as follows. Section 2 covers some key publications on the problem of wireless sensor data acquisition using UAVs. Section 3 formulates the problem. Section 4 describes the architecture and the programming model of GPUs. The modeling of the UAV speed and power consumption is described in Section 5. The details of the proposed framework are presented in Section 6. Finally, Section 7 presents the details and results of the numerical simulations.

## 2. Previous Works

This section presents some key publications that focus on the problem of wireless data acquisition using UAVs. In [3], Na and Yoo present a particle swarm optimization (PSO) algorithm for the optimized positioning of multiple UAVs for the data acquisition from wireless sensors. The algorithm uses a cost function which includes the value of the sensors, the network connectivity between the UAV and the ground station and the communication path quality. One limitation of the proposed approach is that it does not consider the movement of the UAVs between positions, but only the final position of the UAVs. In [4], Pang et al. compare a one-side and a two-side matching algorithms for the data collection and the wireless charging of remote sensors using UAVs. Their problem description assumes that the UAVs return to the base station after visiting each sensor cluster. The utility function used includes the distance between the UAV and the sensor cluster, the amount of data to be collected and the residual energy of the sensors in the sensor cluster. In [5], Chen et al. propose a Direct Future Prediction model to the path planning of a single UAV for the data collection of wireless sensors. They define a value of information (VoI) function which reduces with time following an event and they use a neural network to predict this VoI. Their method also considers the energy of the UAV and guides the UAV towards the charging station when the energy is below a set threshold. They show that their method performs better than Q-learning and Deep Q-Learning. One limitation of their method is the coarse granularity of the environment representation used as they simply divided the search space in a 5 by 5 grid.

In [9], Gong and Shen use dynamic programming to compute the optimal speed for a UAV to collect data from wireless sensors distributed over a line. Their approach can be applied to 2D in the case where a trajectory for the UAV is available beforehand. In [10], Wang et al. decompose the wireless sensor acquisition problem into two sub-problems. The first is essentially the multi-Traveling Salesperson Problem (mTSP) and consists of assigning the wireless sensors to the UAVs and finding in which order they are visited. The second problem consists of minimizing the flight time of each UAV while ensuring that all the data is collected. UAVs are allowed to reduce their speed or hover to ensure sufficient connection time with the wireless sensors. In [11], Yang and Yoo propose a hybrid GA coupled with an Ant Colony Optimization (ACO) for wireless sensor data acquisition by a single UAV. The GA is used to find the order in which the sensors are visited basically solving the TSP and the ACO is used at every step of the GA to calculate paths between sensors. They demonstrated their algorithm on a 10 by 10 discretized map. Their work includes very good descriptions of how GA operators can be adapted to solve the TSP problem.

In [12], Baek et al. present a Lagrange multiplier-based method to solve simultaneously the wireless sensor data acquisition problem and the wireless power transfer problem. One key characteristic of their approach is that they used a transmission radius around the sensor and the UAV does not have to overfly the sensor, but merely enter the circle defined by this radius. This minimizes the overall distance travelled by the UAV. In [13], Kong et al. use a Shuffled Frog-Leaping Algorithm (SFLA) to solve the wireless sensor data acquisition problem considering the maximum transmission range of the sensors. The SFLA uses a PSO-liked optimization on every candidate solution to select the best hovering point within the transmission range of the wireless sensor. In [14], Samir et al. add a constraint to the problem of wireless sensor data acquisition where the data at a wireless sensor must be collected by a UAV before it becomes outdated. They use a branch, reduce and bound algorithm to find the optimal global solution for relatively small-scale scenarios. For larger and more realistic problem sizes, they use convex approximation methods and multiple equivalent transformation to generate suboptimal, but more efficient solutions. Their approximative method relies on the sine cosine optimization algorithm. Their work is limited to a single UAV.

In [15], Chen et al. present a planning algorithm for a UAV to visit and collect data from buoys far off the coast. The buoys are equipped with a power source and are able to recharge the UAV using resonant beam charging. The data collection and trajectory planning problems are formulated as a mixed-integer non-convex optimization problem and is solved using an inexact block coordinate descent method. The solution is also limited to a single UAV. In [16], Ghorbel et al. present a two-step approach which consists of finding the locations of the DPs and then solving the TSP problem. In the first step, they linearize the problem and formulate it as the constrained Weber problem which consists of finding the weighted median of a set of points within a limited area. They use an iterative approach where they solve a set of linear equations until the positions of all DPs converge. In the second step, they solve the TSP with a neighbourhood where the UAV does not have to overfly the DPs, but come in close range. As for the previous work, their solution is limited to a single UAV.

In [17], Baek et al. present a unique method of grouping the sensors nodes into clusters. They use a Voronoi diagram where the edges are located equidistant from the sensors. They then position the DPs at the intersection of the edges so that data from nearby sensors can be downloaded from the DP. They tested their method for a single UAV and showed that the resulting path was shorter than if the UAV had to travel over each sensor node. Finally, in [2], Alfattani et al. present a three-step approach to the wireless sensor data acquisition problem. In the first step, they use a k-means clustering algorithm to find optimized location for the position of ground wireless communication devices responsible for collecting the data from the ground wireless sensors and to relay it to the UAVs. In the second step, they use a GA to solve the mTSP problem so the UAVs visit each one of the ground wireless relays and return to the base station. In the third step, they use a geometrical model to adjust the path of the UAVs to fly to the neighborhoods of the relay points instead of directly above the relay point in an effort to minimize the overall distance travelled by the UAVs.

There are four main limitations to previous methods that are addressed in this paper. First, the approaches surveyed all tackle the problem of sensor data acquisition in a 2D environment. To be used in a realistic setting, the method must allow for a 3D environment as it is done in this paper. Secondly, environment representation is often very small, this is obvious in [5] where the environment is represented using a 5 × 5 grid or a 10 × 10 grid in [11]. Thirdly, very few publications actually compute the path between DPs. Most resort to using straight lines. In this paper an SSSP algorithm is used to compute optimal paths between the DPs. Lastly, which is also related to the size of the environment representation, very few publications comment on the runtime of their algorithm. As sensors are discovered, the algorithm must be able to recalculate paths in a relatively short time. In this paper, the SSSP is parallelized on a GPU architecture to speed up the overall framework by a factor of 33.3× and accelerate the calculation of the trajectories.

## 3. Problem Formulation

The problem formulation assumes a wireless network composed of a set S of sensors positioned on the ground and a set U of UAVs positioned in the air. The sensors are low-power and the UAVs must come into close range and in line of sight to download the data from them. All communications are assumed to be orthogonal. The terrain is modelled using a 3D surface and the airspace includes no-fly zones that the UAVs must avoid. After being polled by UAV u, sensor s transmits its data. Following the model presented in [2], the received Signal-to-Noise Ratio (SNR) $\gamma_{s,u}$ by UAV u, can be computed as follows.

(1) $$\gamma_{s,u}=\frac{p_{s\;}d_{s,u}^{-\alpha }}{\sigma^{2}}$$

where $P_{s}$ is the power of the signal emitted by sensor s, $d_{s,u}$ is the distance between sensor s and UAV u, α>0 is the path-loss exponent and $\sigma^{2}$ is the noise power. The communication link is successful if $\gamma_{s,u}\geq \gamma_{th}$, where γth is the SNR threshold. Accordingly, a maximum communication range can be defined as follows:

(2) ds,u≤ dth=(psσ2γth)1α

The following notation shall be used:

- T to represent the region of space under the surface of the terrain;
- NF to represent the region of space that corresponds to the no-fly zones;
- $U=\left\{u_{i}:\;i=1\ldots N_{u}\right\}$ to represent the set of UAVs;
- $W_{u}=\{W_{u,i}\;|\;i=1\ldots N_{u,W}\}$ as the set of waypoints that defines the trajectory of UAV u;
- $v_{u,i}=$ the average speed of UAV *u* between its waypoints $W_{u,i}$ and $W_{u,i+1}$;
- $S=\left\{s_{i}:\;i=1\ldots N_{s}\right\}$ as the set of sensors, positioned on the ground at the coordinates $\left\{s_{i}\right\}$;
- $D_{s}=$ the amount of data to be transferred by sensor s;
- $r_{s}=$ the rate of transfer of the data by sensor s;
- $DP=\{p_{i}\;|\;i=1\;\ldots \;N_{DP}\}$ as the set of download points, with the coordinates $\left\{p_{i}\right\}$;
- $DP_{u}=\{p_{u,i}\;|\;i=1\ldots N_{u,DP}\}$ as the set of download points from which UAV u will gather data; and
- $S_{p}=\{\;s\in S\;|\;d_{s,p}\;\leq \;d_{th\;}\}$ the set of sensors that can be polled from download point p.

In this work, it is assumed that all sensors transmit the same amount of data, at the same rate, have the same transmission range and that at any given download point $p_{u}$, UAV u successively gathers data from every sensor that is in range. Thus, the time required by UAV u to download all of its data depends on the number of sensors associated with the download point and the transfer rate. The download time and the travel time for UAV u in this mission, is therefore

(3) $${𝒯}_{u,download}=\sum_{p\;\in \;DP_{u}}^{}\left\{\sum_{s\;\in \;s_{p}}\frac{D_{s}}{r_{s}}\right\}\;$$

and

(4) $${𝒯}_{u,travel}=\sum_{i=1\ldots N_{u,w}}^{}\frac{∥W_{u,i+1}-W_{u,i}∥}{v_{u,i}}$$

The problem of wireless sensor data acquisition using a team of UAVs can now be expressed as that of minimizing the maximum time taken by any UAV to perform its mission, i.e., finding

(5) $$\underset{\begin{array}{c} \;S_{p}\;\mathrm{with}\;p\;\in \;DP\\ DP_{u}\;\mathrm{and}\;W_{u}\;\mathrm{with}\;u\;\in \;U \end{array}}{\min}\;\;\left\{\underset{u\;\in \;U}{\;\max}\;\left[{𝒯}_{u,travel}+{𝒯}_{u,download}\right]\right\}$$

subject to the following constraints:

- $\left(s,\;p\right)\cap T=\emptyset \;\forall s\in S_{p}$ and p∈DP because the reading of the sensor must be made in line of sight,
- $DP_{u}\cap DP_{v}=\emptyset \;\forall u\neq v\in U$, so that two different UAVs will not read sensors at the same download points,
- $U_{u\;\in \;U}DP_{u}=DP$ so that all the download points are read by some UAV,
- $S_{p}\cap S_{q}=\emptyset \;\forall p\;\neq \;q\in DP$, so that the sensors read at two different download points are all different,
- $U_{p\;\in \;DP}S_{p}=S$ so that all the sensors are read at some download point,
- $DP_{u}\subseteq W_{u}$ ∀u∈U, so that all the download points for UAV *u* are some of its waypoints, and
- $\left(W_{u,i},W_{u,i+1}\right)\cap T=\emptyset$ and $\left(W_{u,i},W_{u,i+1}\right)\cap NF=\emptyset$ $\forall i=1\ldots \left(N_{u,w-1}\right)$ and ∀u∈U, so that no trajectory leg traverses the terrain nor a no-fly zone.

As defined in Equation (5), the problem consists of finding the location of the download points *p_i_*, assigning the sensors to the $p_{i}$, assigning the download points to the UAVs and calculating the trajectory for each UAV in order to minimize the total mission time that is the flying time of all UAVs together with their data transfer time. One can note that minimizing the overall mission time results from minimizing the maximum mission time of all UAVs. Although here, Equation (5) is designed to minimize the overall time of the mission, the proposed framework is configurable and can be used to minimize the distance or energy consumption depending on the mission parameters chosen by the user.

## 4. GPU Architecture

**Figure 1:**
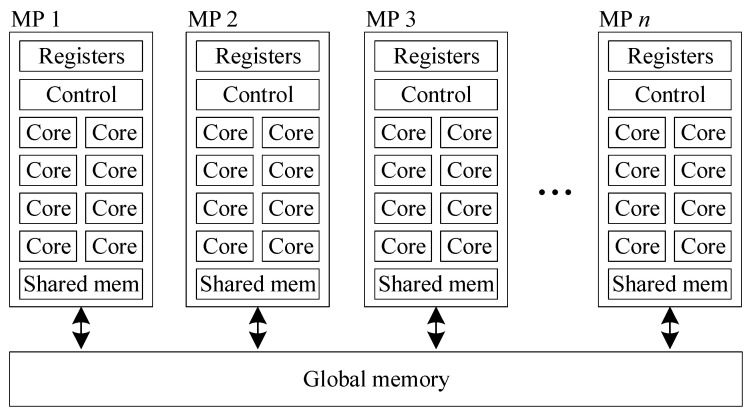
Simplified architecture of an NVIDIA GPU.

The architecture of GPUs has evolved over the last two decades from fixed pipelines to programmable processors which has extended their use to scientific applications or in fact any application that exhibits a high level of data parallelism. In this paper, the GPU is used to speed up the SSSP algorithm and find optimal paths between the DPs much faster. The GPU used is an NVIDIA RTX 2080 Ti [18]. A simplified diagram of the architecture of NVIDIA GPUs is shown in Figure 1. The GPU is composed of a large number of cores grouped into multiprocessors (MP). Each core has its own registers, but share the control logic and the shared memory with the other cores in the same MP. The GPU also has a global memory which is shared by all the cores across all the MPs. The RTX 2080 Ti has 4352 cores, 68 MPs, 64 K registers, and 64 KB of shared memory per MP. The global memory is 11 GB in size and has a bandwidth of 616 GB/s which is much faster than that of a typical CPU. The cores of the RTX 2080 Ti operates at a base frequency of 1350 MHz and a boost frequency of 1545 MHz producing 13.45 TFlops of computing power.

NVIDIA GPUs can be programmed using the CUDA language which is an extension to the C and C++ languages. It allows the programming of kernel functions which are parallel functions executed on the GPU. When launching a kernel, the threads are divided into thread blocks. Each block is mapped to a SM. Threads in the same thread block can share data using the shared memory, but threads from different thread blocks cannot communicate between themselves. The only way to synchronize or share data across the thread block is to launch a new kernel. Although there are a large number of cores per SM, there is only a limited number of control logic units. This means that maximum performance is achieved when all the threads within a same SM execute the same instruction at the same time. If thread takes different branches of an if statement, referred to as thread divergence, branches are executed sequentially.

## 5. UAV Model

### 5.1. Speed and Power at Steady State

The proposed framework can be configured to minimize distance travelled, mission time, or energy consumption. For the last two objectives, a model must be used to estimate the speed of the UAV and its energy consumption on each segment forming the path. Several papers define energy models for quadcopters based on the aerodynamic and electric properties of the vehicles such as [19,20,21,22]. The disadvantage of using such model is that it does not consider the autopilot and the UAV as a single system. If the autopilot is not able to fly the trajectory exactly as described, the energy predicted is not correct. A method that is favored by the author of this present paper is to rely on experimental flights to model the UAV and autopilot as a single black box. The model used here is for the IRIS quadcopter equipped with the PX4 autopilot and was developed by Di Franco and G. Buttazzo in [23]. To find the optimal speed at steady state during horizontal movement, the authors measure the power consumed at multiple speeds and calculate the energy per distance travelled in J/m. The lowest value is achieved at optimal speed. For horizontal movement, they find an optimal speed and power consumption of 12.5 m/s and 244.2 Watts. For vertical ascent and vertical descent, the authors find that the UAV/autopilot system travels at a speed of 2.08 m/s with a power consumption of 249.1 Watts when ascending and 1.28 m/s and 212.5 Watts when descending. In this work, optimal speed and power at steady state are used to set the edge weights when building the graph for the SSSP algorithm.

### 5.2. Acceleration and Deceleration

**Figure 2:**
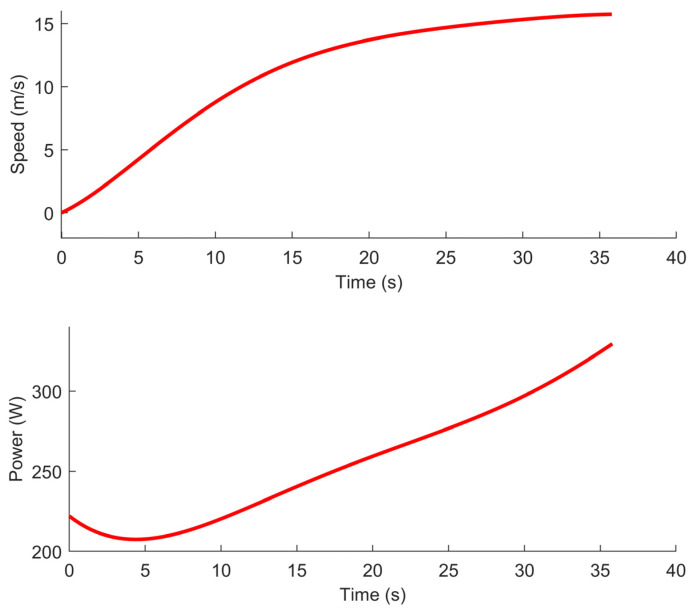
Sixth degree interpolation of the speed and power consumption versus time at maximum acceleration for the IRIS quadcopter with the PX4 autopilot. Calculated using the data published in [23].

To account for acceleration and deceleration, Di Franco and G. Buttazzo [23] measure the speed and power of the UAV/autopilot versus time during maximum horizontal acceleration and deceleration. They then use a 6th degree polynomial interpolation to model the data. In this paper, the same approach is used. The data from the figures published in [23] was digitized using the GetData [24] software and used to calculate the 6th degree polynomial interpolation using MATLAB. The inverse of the data was also used to calculate the 6th degree polynomial interpolation for the time versus speed. This inverse function comes very handy later on to find the time at which a given speed is achieved. The polynomial interpolation obtained from the data published in [23] are shown in Figure 2. Although not shown here, similar graphs exist for the speed and power consumption during deceleration from maximum speed to zero. As for the acceleration, the interpolation for the time versus speed during deceleration was calculated. In this work, the acceleration and deceleration models are used to precisely calculate the speed and energy consumption of the UAV on the final path.

### 5.3. Speed Reduction When Turning

**Figure 3:**
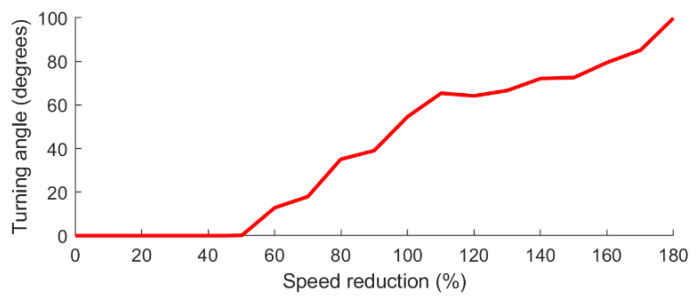
Speed reduction when negotiating a turn at various angles as measured in [25].

In their paper [23], Di Franco and G. Buttazzo assume that the UAV/autopilot system comes to a full stop before changing direction which is an overly conservative estimate of the speed and power consumption. In a subsequent publication [25], they note that in reality the UAV/autopilot system reduces its speed before engaging into a turn, but does not come to a full stop unless the turn is a 180-degree turn. Based on this observation, they refine their model and find during experimental trials that the speed reduction in percent for the UAV/autopilot is about the same for a given angle no matter what the incoming speed is. In this paper, the data measured in [25] is used as part of the UAV model. Again here, the data is taken from the graph published in [25] and digitized using the GetData [24] software. The speed reduction in percent for the IRIS quadcopter for various turning angle is shown in Figure 3. This data is stored in a lookup table and used by the proposed framework when assigning the penalties for changing direction in the SSSP graph and also when calculating the speed of the UAV on the final path. The details of this calculation are given in Section 6.6.

## 6. Material and Method

### 6.1. Calculating the Location of the Download Points

The first step of the proposed algorithm is to calculate the *x*, *y*, *z* position of the DPs where the UAVs will position themselves to download the data from the wireless sensors. This is done by using an iterative approach that relies on the k-means clustering algorithm. The pseudocode of the procedure is given in Algorithm 1. It starts by running the k-means with a single cluster. Although the sensors have a 3D position (they are located on the ground), the k-means is run in 2D, this is to avoid finding a centroid below the elevation of the ground. Once the centroid of the cluster has been found, its elevation is set based on the elevation of the terrain and the default flying altitude of the UAVs. Then, for the cluster found, the distance between the centroid and each sensor is verified. If this distance is longer than the range of the wireless sensors, a new centroid is positioned at the location of the sensor and the algorithm goes immediately back to running the k-means without checking the other sensors. Similarly, it checks to see if the centroid and the sensor are in line of sight and if they are not, a centroid is added at the sensor and the k-means is called again. This process is repeated until all the wireless sensors are in range and in the line of sight of their centroid.
**Algorithm 1.** Calculation of the positions of the download points1: Initialize the k-means to use one centroid at the center of the map2: Run k-means in 2D3: Add elevation to the centroid(s) based on the terrain elevation4: **for each**
*cluster*
**do**5:   **for each**
*sensor in cluster*
**do**6:     **if** distance between centroid and sensor is longer than wireless7.:     range **or** centroid and sensor are not in line of sight8:        Add a new centroid at (*x*, *y*) location of the sensor9:        **goto** line 210: Return the location (*x*, *y*, *z*) of the centroids

**Figure 4:**
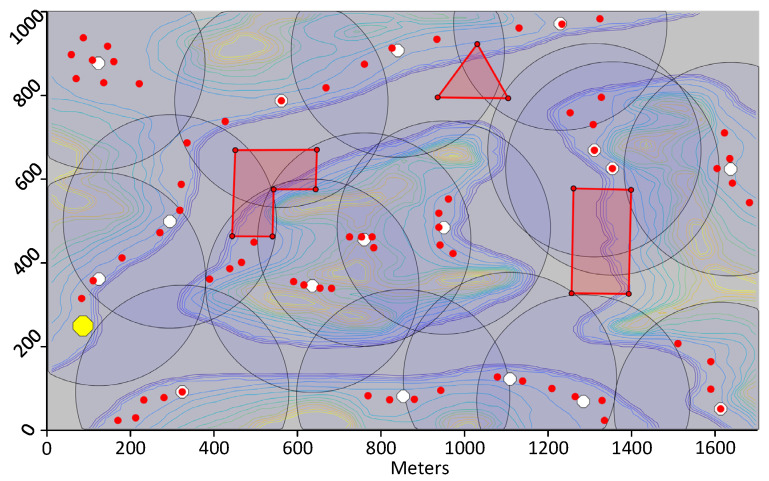
1700 by 1000-m map showing 70 sensors in red strategically positioned, 15 download points in white and the starting point of the UAVs in yellow. There are also three no-fly zones represented by the red polygons.

The iterative k-means approach used is efficient, but may add more clusters than necessary. To account for this, a function was added which is described at Algorithm 2 to remove unnecessary clusters. This function checks each cluster and each sensor within each cluster. If all the sensors in a given cluster are within the range and in the line of sight of another cluster, the cluster can be removed. To demonstrate the functioning of the calculation of the DPs, 70 sensors that have been strategically positioned on a 1700 by 1000-m map are shown in Figure 4. This artificial map represents a canyon with a river, an island and some mountains. The altitude varies from 0 to 250 m. The map also contains three no-fly zones represented using red polygons. The sensors have a wireless transmission range of 170 m. When running Algorithm 1 to calculate the location of the DPs, 22 DPs are calculated. Following the reduction function at Algorithm 2, 15 DPs are remaining clearly showing the need for the removal of extra DPs.
**Algorithm 2.** Removal of extra download points1: **for each**
*cluster i*
**do**2:  *Nb_sensors_covered_by_centroid_i* = 0;3:  *Nb_sensors_covered_by_other_centroids* = 0;4:  **for each**
*sensor in cluster i*
**do**5:     *Nb_sensors_covered_by_centroid_i ++*;6:     **for each**
*cluster j*
**do**7:        **if**
*i*
**not equals**
*j*
**do**8:           **if** distance between centroid *j* and sensor is smaller than9:           wireless range and centroid *j* and sensor are in line of sight **do**10:            *Nb_sensors_covered_by_other_centroids ++*;11:  **if**
*Nb_sensors_covered_by_centroid_i*
**equals**
*Nb_sensors_covered_by_other_centroids*
**do**12:    Remove centroid;

### 6.2. Building the Graph

**Figure 5:**
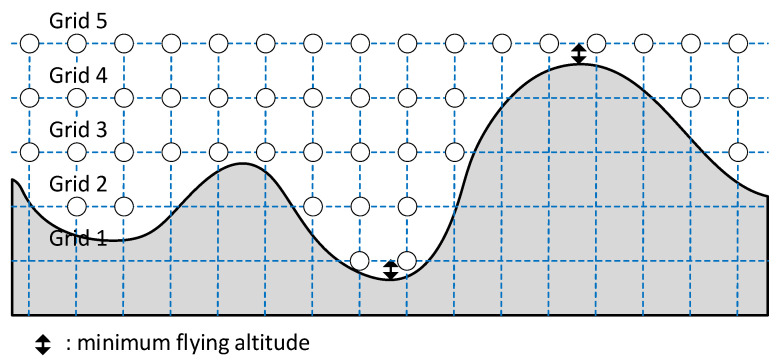
Five grids positioned above the 3D terrain.

In this stage of the framework, the environment is discretized as a vertical superposition of horizontal grids of nodes above the 3D terrain. The nodes represent the position that can be occupied by the UAVs whereas the edges between the nodes represent the line segments that can be travelled by the UAVs. The number of superposed grids $N_{g}$ is configurable and set by the user, and all grids are vertically equally spaced. In all cases, the highest grid is positioned at $Z_{max}$, the highest elevation of the terrain plus the minimum flying altitude of the UAV. The lowest grid is positioned at $Z_{min}$, the lowest elevation of the terrain plus the minimum flying altitude of the UAV. If there are to be $N_{g}$ grids stacked between the two limiting altitudes, the spacing between them has to be $\Delta H=\left(Z_{max}-Z_{min}\right)/N_{g}$. An example for five grids is illustrated in Figure 5.

**Figure 6:**
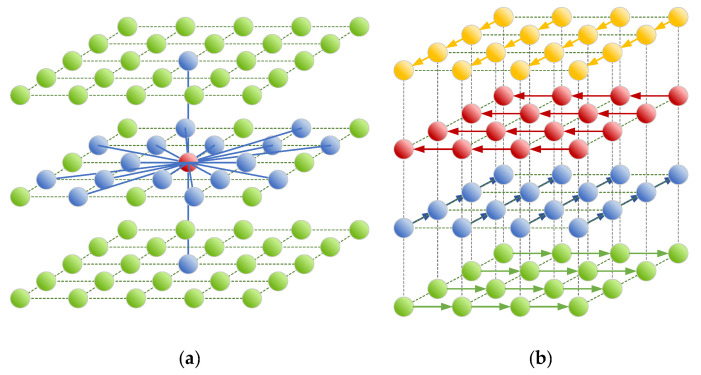
(**a**) Grid showing the connection between a node and its 18 neighbors. (**b**) Representation of the multiple grids superposed in the same plane where each grid allows travel in a single direction.

Each node in the proposed algorithm is connected to its 18 neighbors, 16 on the same plane, the one directly above and the one directly below, as shown in blue in Figure 6a. The 16-neighbor topology within the horizontal plan was chosen because it allows for more directions of travel compared to a 4-neighbor or an 8-neighbor topology which only connects a node to its immediate neighbors. Having more directions of travel allows for better paths between DPs. The weights of the edges connecting the nodes are set based on the optimization objective used and can be the distance between the nodes, the time needed to travel the edges at optimal speed or the energy required to travel the edges, also at optimal speed. The result is a connectivity graph that is used at the next step by the SSSP algorithm to compute optimal paths connecting every pair of DPs.

One drawback of using a graph built as explained so far is that there is no way for the SSSP algorithm to minimize the number of turns. It is common for such algorithm to generate a zig-zag type of path which is much less efficient to fly than a straight path. In order to minimize the number of turns in the path, the technique described in [26] is implemented. The technique consists of overlaying multiple grids at the same altitude. On each grid, the UAV is allowed to travel in a single direction. At any points on a grid, the UAV is allowed to change grid by incurring a penalty. This is illustrated in Figure 6b for only four directions. In this work, there are 18 grids overlaid at every altitude where there used to be a single grid. On a grid, a node is connected to a single neighbor located physically at the different location. However, at a same location, a node is connected to the 17 other nodes belonging to the 17 other grids that are overlaid. To calculate the cost associated with changing grid, i.e., changing direction, the time, or energy needed to reduce the speed before taking the turn is calculated.

For the case of going from a horizontal segment to another horizontal segment, the values plotted in Figure 3 are used to determine the speed reduction in percent. The incoming speed is assumed to be the optimal speed of the UAV. Based on the percent value, the actual reduced speed can be calculated. Assuming the deceleration before the turn starts at $t_{1}=0$, the polynomial interpolation for the inverse time vs speed function is used to get $t_{2}$, the time at which the UAV reaches the reduced speed. The same approach is used to find $t_{3}$, the time during acceleration where the UAV is at the reduced speed, and $t_{4}$, the time when the UAV reaches back optimal speed. The time used for the deceleration and acceleration are then calculated as follows:

(6) $$t_{a}=\left(t_{2}-t_{1}\right)+\left(t_{4}-t_{3}\right)$$

By evaluating the integral, which is quite easy due to the speed being modeled as a polynomial function, the distance traveled by the UAV during the deceleration and acceleration can be calculated using Equation (7). The same can be done for the energy consumption using Equation (8).

(7) $$D_{a}=\int_{t_{1=0}}^{t_{2}}S_{dec}\left(t\right)+\int_{t_{3}}^{t_{4}}S_{acc}\left(t\right)$$

(8) $$E_{a}=\int_{t_{1=0}}^{t_{2}}P_{dec}\left(t\right)+\int_{t_{3}}^{t_{4}}P_{acc}\left(t\right)$$

To calculate a penalty cost associated with the turn, the time, and energy needed to travel this same distance, but at optimal speed are calculated as follows as a reference.

(9) $$t_{b}=D_{a}/v_{optimal}$$

(10) $$E_{b}=t_{b}\;P_{optimal}$$

The penalty costs are then calculated by subtracting this second value from the first one:

(11) $$t_{cost}=t_{a}-t_{b}$$

(12) $$E_{cost}=E_{a}-E_{b}$$

The values $t_{cost}$ and $E_{cost}$ are the weights used in the connectivity graph to change direction between two horizontal segments. For the case of going from a horizontal segment to a vertical segment, the same logic is followed, but the reduced speed is set to zero and only the deceleration terms are used in Equations (7) and (8). This means that the model used here assumes that the UAV comes to a full stop before ascending or descending and that the time used for vertical acceleration is negligible. For the case of going from a vertical segment to a horizontal segment, the reduced speed is set to 0 and only the acceleration terms in (7) and (8) are used.

The building of the graph is done using nested for loops and an iterator in a way to directly produce a graph in the Compressed Sparse Column (CSC) format without further processing. The CSC sparse format is used at the next step by the Dijsktra algorithm implemented in the C++ Boost library. However, for the CUDA implementation, it is the Compressed Sparse Row (CSR) format that is used. For this reason, we generate the CSR graph from the CSC representation.

### 6.3. Single Source Shortest Path Algorithm

The SSSP algorithm calculates the shortest path between a starting node and every other node in the graph. This means that to calculate the shortest path between a DP and every other DP, the SSSP only needs to be run once. It also means that to calculate the shortest paths linking any two DPs in a set of *N* DPs, *N* instances of the SSSP algorithm need to be run. Depending on the weights assigned to the edges of the graph, the final path may minimize distance, time, or energy. In the proposed framework, two implementations of the SSSP algorithm are used. The first one is the Dijkstra algorithm provided by the C++ Boost library [27]. This implementation is regarded as highly efficient and is used here as the reference for the sequential program. The second implementation is an improved version developed by the author of this paper of the parallel SSSP algorithm proposed by Harish et al. [28] for the GPU architecture. As it was shown in a previous publication [29], this adaptation is more performant than other implementations on GPU recently published in the literature [30,31]. This second implementation is preferred due to its shorter execution time which allows for recalculation of the paths in case new obstacles or new sensors are discovered. The details of Harish et al.’s algorithm are presented at Algorithms 3–5. Algorithms 3 and 5 are from the original algorithm developed by Harish et al. while Algorithm 4 is the improved version proposed in this paper.

The SSSP algorithm keeps an array of frontier flags *F*, of cost *C* and of updated cost *U* of size *n* where *n* is the number of nodes in the graph. The array *F* identifies the nodes whose weights have been updated in an iteration and that must be visited in the next iteration. These nodes are said to be at the frontier. The array *C* keeps the cost of the node, i.e., the cost needed to travel from the start node to the node. The array *U* is used to update the nodes cost synchronously at each iteration. Initially, the *F* array is set to false, the *C* array and the *U* array are set to infinity. At line 2 of Algorithm 3, the flag for the start node is set to true and its cost and updated cost are set to 0. A sum value which represents the number of nodes at the frontier is set to 1. The algorithm then goes into a while loop for as long as there are nodes in the frontier. The loop is composed of two kernels (parallel functions in CUDA). In the first kernel, all the nodes are visited in parallel using one thread per node. Nodes that are at the frontier have the weight of their neighbors updated as needed. This update operation creates a race condition as multiple threads could be updating the same node. In the original algorithm, an atomic operation was used by Harish et al. [28], but this operation slowed down the execution of the algorithm. In the proposed implementation, the atomic operation is avoided by forcing the source node *tid* to remain in the frontier node so that it is revisited at the next iteration. This means that if the value it wrote to *U*[*i*] was overwritten by another thread, it would rewrite the value to *U*[*i*] at the next iteration. This necessarily generates more iterations, but avoids the atomic operation. Overall, a faster execution was observed by avoiding the atomic operation. In the second kernel, all nodes are visited and any nodes whose cost was updated have their flag set to true. These nodes are now part of the frontier and will be visited at the next iteration. After kernel 2 finishes, a parallel reduction is used to calculate the number of nodes in the frontier and the algorithm proceeds to the next iteration of the while loop. After the SSSP algorithm terminates, the cost array is transferred from the GPU to the CPU. The cost arrays for all instances of the SSSP are kept in memory for the path reconstruction phase explained in Section 6.6.
**Algorithm 3.** Harish SSSP (***G***, ***s***, *e*) [28]1: Initialize all ***F*** to false, all ***C*** to ∞ and all ***U*** to ∞2: Set ***F***[***s***] = true, ***C***[***s***] = 0, ***U***[***s***] = 0, *sum* = 13: **while**
*sum* > 0 **do**4:   kernel 1 (***G***,***F***,***C***,***U***) with 1 thread per node5:   kernel 2 (***F***,***C***,***U***) with 1 thread per node6:   *sum* = reduce_sum (***F***)7: Build path backward starting at vertex *e* using ***G*** and ***C***

**Algorithm 4.** kernel 1 (***G***, ***F***, ***C***, ***U***) [proposed implementation]1: **for all** node *tid*
**in parallel do**2:   **if**
***F***[*tid*] **then**3:      ***F***[*tid*] = false4:      **for all**
*to*-neighbors *i* of *tid*
**do**5:         **if**
***U***[*i*] > ***C***[*tid*] + ***W***[*i*, *tid*] **then**6:            ***U***[*i*] = ***C***[*tid*] + ***W***[*i*, *tid*]7:            ***F***[*tid*] = true

**Algorithm 5.** kernel 2 (***G***, ***F***, ***C***, ***U***) [28]1: **for all** node *tid*
**in parallel do**2:   **if**
***C***[*tid*] > ***U***[*tid*] **then**3:     ***C***[*tid*] = ***U***[*tid*]4:     ***F***[*tid*] = true5:   ***U***[*tid*] = ***C***[*tid*]

Two optimizations were done in the proposed CUDA implementation to further improve its performance. First, as an SSSP instance is solved for each pair of DPs, the SSSP instances are solved in batch. This improves the level of parallelism exploited, but also increases the amount of memory needed on the GPU so care must be taken not to use a batch size that is too large. It was found through experimentation that solving 10 SSSPs simultaneously was a good compromise. To keep track of which batches are active and have not converged yet, an array of indices that is compacted at every iteration of the while loop is kept. This compaction is done on the GPU using the CUDA Cub library [32]. Because multiple batches are processed concurrently, the sum reduction operation in Algorithm 3 must be converted to a segmented sum, also provided by the CUDA Cub library.

Although performed in parallel, the compaction operation and the segmented sum operation are quite costly. A second optimization is to do multiple iterations of the while loop in Algorithm 3 before doing the compaction and the segmented sum. The while loop executes extra few iterations at the very end, but the benefit of skipping the compaction and the segment sum is far superior. The performance improvement of those two optimizations is measured in Section 7.2.

### 6.4. Building the Cost Matrix

Once the optimal path connecting each pair of DPs have been computed, the next step is to build the cost matrix that is used by the GA at the next step. An example of a cost matrix for 10 DPs is shown at Table 1. It contains the cost of going from every DP to every other DPs. When optimizing for distance, filling the cost matrix is quite simple. The weight of the final node for each instance of the SSSP algorithm is simply taken. The resulting matrix is symmetrical. When optimizing for time or power consumption, the process is more complicated and requires that each path be rebuilt to accurately compute the time and energy required to travel the path. Although this operation is done at this step for every pair of DPs, the description of the path reconstruction is left to Section 6.6. The time and energy used to hover at the DP are also added to the cost of the path.

### 6.5. Genetic Algorithm

Once the cost matrix is built, the GA is used to assign the DPs to the UAVs and to find the order in which they will be visited forming complete tours. The GA is a metaheuristic that was first proposed by John Holland in 1975 [33]. As of today, it is still one of the most broadly used metaheuristics in engineering. The pseudocode is given at Algorithm 6. It works by creating a population of candidate solutions referred to as the parent solutions. It then starts an iterative process where parent solutions are selected, child solutions are created from good parents using crossover, child solutions are modified using mutation and parent solutions are replaced by the child solutions before the next iteration. Once the termination criterion has been met, the best solution is returned by the algorithm.
**Algorithm 6.** Genetic Algorithm1: Initialize the parent solutions2: **while**
*iteration* < *max iteration*
**do**3:   compute fitness of parent solutions4:   select parent solutions through selection5:   create child solutions through crossover6:   modify child solutions through mutation7:   replace parent solutions8: Return best solution

**Figure 7:**
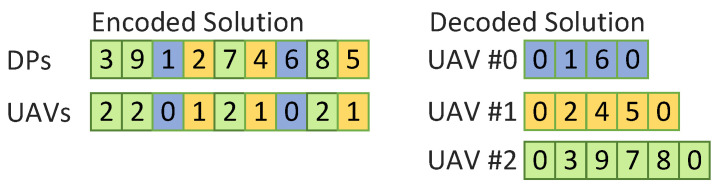
Solution encoding.

**Figure 8:**
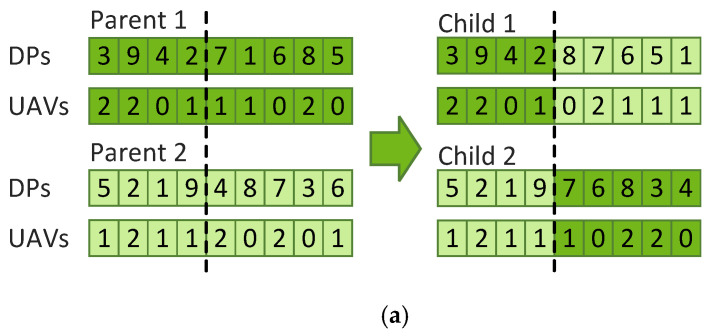

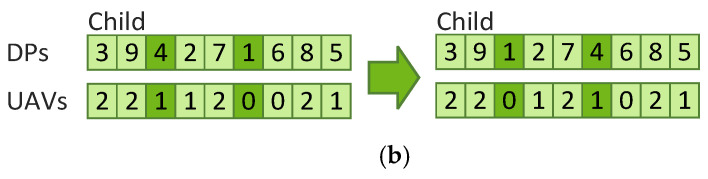
(**a**) Crossover operator and (**b**) mutation operator.

In our implementation, the candidate solutions are encoded as illustrated in Figure 7. The solution is represented using two strings where the first one includes the DPs visited by the UAVs and the second one includes which UAV visits each one of the DPs. When decoding the solution strings, the start point (i.e., point 0) is added at the beginning and at the end of each route resulting in a tour. The GA is configured to use 100 candidate solutions with 200 iterations. These settings were tested experimentally and generated good results. The selection is made by tournament where two parents are randomly picked and the best one is retained. The process is repeated to select the second parent. The crossover is done using a single-point crossover as shown in Figure 8a. The crossover point is selected randomly, the elements before the point come from the first parent while the elements after the points come from the second parent. When selecting the elements from the second parent, one must be careful not to select elements that have already been selected from the first parent. Once the end of the second parent has been reached, the selection starts back from the beginning of the second parent. The inverse is done to create child 2. The mutation operation is implemented as illustrated in Figure 8b where two elements from the solution strings are selected and swapped. Not shown in Figure 8, but applied to every child solution is the 2-opt local search heuristic which removes loops from the solution. The readers are referred to [34,35] for more details on the 2-opt local search. Finally, the replacement is implemented with elitism where the two best parents survive to the next iteration and replace the two worse children. Being a non-deterministic algorithm, the GA may produce different results every time it is run, to maximize the quality of the final path found, the proposed framework runs the GA three times and selects the best solution from the three runs.

### 6.6. Building the Final Path

Using the route for each UAV (i.e., the series of DPs) and the nodes weight C from the SSSP instances, the final path can be built by constructing the trajectories connecting the DPs together. To build the trajectory from the SSSP instances, one must start from the final node and find the previous node, using the CSC graph representation, whose weight plus the edge weight is the smallest. The process is repeated until the start node is found. During this process, the direction of the edge leading to the previous node is calculated and the node is added to the path only when there is a change in direction. This allows for the creation of a path with the minimum number of nodes.

Once the path is built, the speed, power consumption, and position of the UAV as a function of time are calculated precisely. For this, the UAV model discussed in Section 5 is used. For vertical segment, the acceleration and deceleration are ignored and the UAV speed and power are set to their optimal values. For horizontal segments, the segment can be decomposed into three sections: the acceleration, the steady state, and the deceleration. Starting with the first segment of the path, the initial speed is necessarily zero. Based on the angle with the next segment the speed reduction as a percent of the maximum speed reached on the segment is determined by looking in the lookup table discussed in Section 5. A binary search is then used to find the value of $v_{max}$, the maximum speed reachable on the segment. The binary search is bound by 0 and $v_{optimal}$, the optimal speed of the UAV. A speed is reachable when there is enough distance to accelerate to the speed and enough distance to decelerate to the reduced speed. If $v_{max}$ is equal to $v_{optimal}$, the segment is necessarily composed of the three sections (i.e., acceleration, steady state, and deceleration). If $v_{max}$ is smaller than $v_{optimal}$, there is no section at steady state. When calculating $v_{max}$, the start and end time for the acceleration, the steady state and the deceleration are also computed. The polynomial interpolation functions for the acceleration and deceleration are used to set the instantaneous speed and power of the UAV. The position of the UAV is calculated based on the previous position and the instantaneous speed. The speed at the end of the segment is used as the initial speed for the next segment and the process is repeated.

In the event that the initial speed at the beginning of a segment is too high to accommodate for a deceleration to the reduced speed found in the lookup table for a given turning angle, the initial speed that would allow the deceleration is calculated and used as the final speed for the previous segment. The final speed for the previous segment becomes a constraint and the previous segment is reprocessed. This can be repeated multiple times allowing the algorithm to revisit multiple previous segments. This can happen in a situation where there is a long segment followed by a series of short segments followed by a sharp turn. The UAV would achieve a high speed on the long segment, would not reduce its speed before entering the short segments and would not have enough distance to reduce its speed before arriving to the sharp turn. With the proposed approach, the UAV will reduce its speed on the long segment and will arrive at the correct speed at the sharp turn.

### 6.7. Collision Avoidance

After the final path for each UAV has been constructed, the array *position* which contains the *x*, *y*, and *z* position of the UAV at each time increment, the array *speed* which contains the speed of the UAV at each time increment and the array *is_stationary_point* which identifies if the position is a DP or the starting point are available. The time increments for each UAV are the same which means that *position*[*i*] for UAV #1 and *position*[*i*] for UAV #2 represent the position for both UAVs at the exact same time. The collision avoidance function checks each pair of UAVs for a possible collision. It iterates through the position array of the two UAVs and calculates the distance between them. If the distance is below a set threshold and none of the UAVs is currently at the starting point, a collision has been detected. The condition for a UAV at the starting point is there as multiple UAVs are allowed to be at the starting point at the same time which is also the charging station. Once a collision has been detected at *position*[*i*]. The function uses the *is_stationary_point* array and decrement *i* until the previous DP or the starting point is reached. The function then inserts values in the *position*, *speed* and *is_stationary_point* arrays to force the UAV to wait longer at the DP. Once this is done, the function restarts the check for collision for every pair of UAVs. This process is repeated until no collisions are detected.

## 7. Numerical Simulation and Results

### 7.1. Test 1: Calculating Trajectories

**Figure 9:**
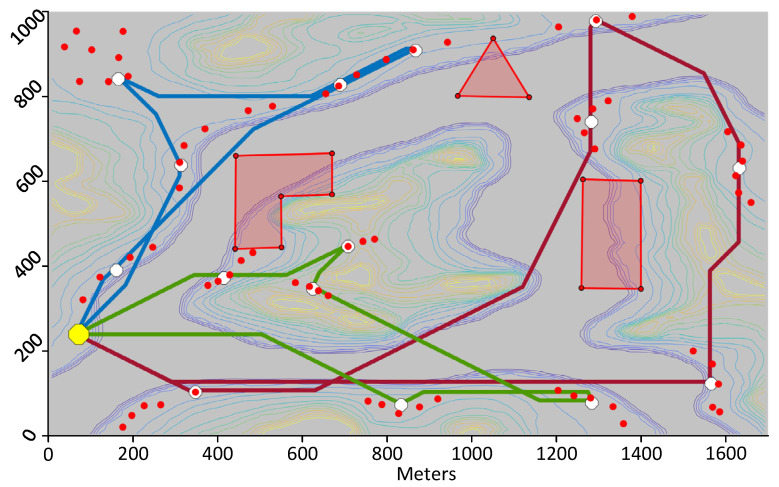
Paths minimizing distance for three UAVs visiting 15 DPs covering the 70 sensors.

**Figure 10:**
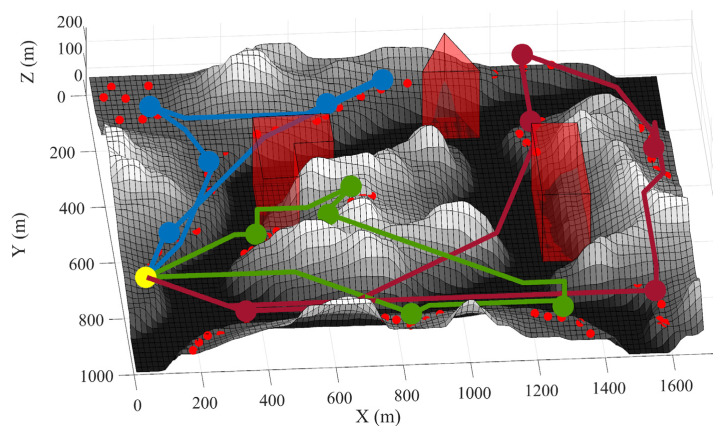
Same paths as in Figure 9, but shown in 3D.

**Figure 11:**
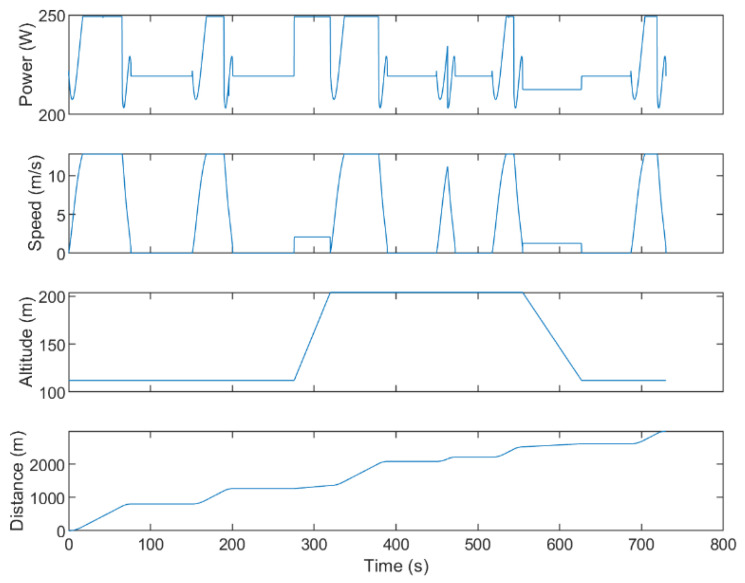
Flight profile for the UAV in green in Figure 9.

The first test consists of using the proposed framework to compute trajectories for UAVs that visit the DPs whose location was calculated as per Section 6.1. The first trial uses the same map and DPs shown in Figure 4. The calculated trajectories that minimize distance are shown in 2D in Figure 9 and in 3D in Figure 10. When minimizing distance, the distance of the longest path is minimized. It can be observed that all DPs are successfully visited and that the no-fly zones are avoided. The flight profile for the green UAV is plotted in Figure 11. The UAV flies the path counter-clockwise starting at the yellow dot. The UAV takes off and climbs to the minimum flying altitude to reach the starting point. This takeoff is not shown in the flight profile. The UAV then flies at constant altitude and visits the first two DPs. On the speed diagram, one can see that the UAV hovers at the DPs to download the data from the sensors. The UAV then immediately climbs to the altitude of the next two DPs and flies to the third DP reaching steady speed. This may be easier to see on the 3D diagram. After hovering at the third DP, the UAV accelerates, but does not have a sufficient distance to reach steady speed and must decelerate to stop at DP 4. After DP 4, the UAV flies and stops at a high altitude above DP 5. It then descends until it reaches the proper altitude to download the sensor data. After downloading the data, the UAV returns to the starting point at constant altitude. The recovery of the UAV is not shown on the flight profile. The statistics for the three paths are listed in Table 2.

**Figure 12:**
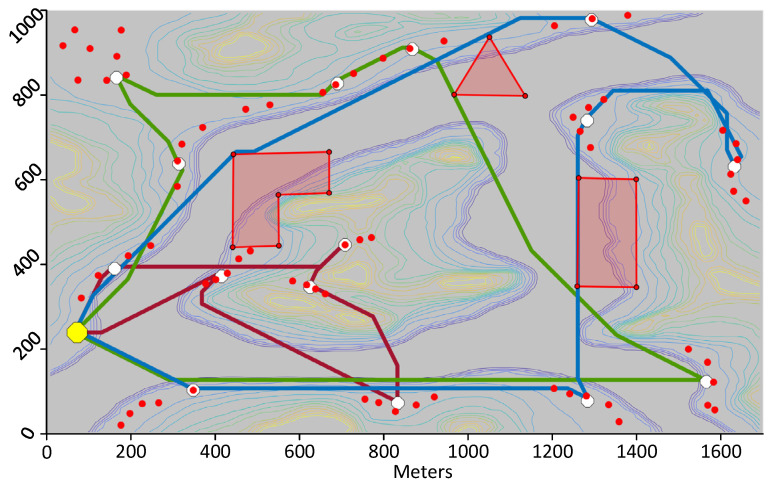
Path minimizing time for three UAVs visiting 15 download points covering the 70 sensors.

The second trial uses the same scenario, but consists of minimizing time instead of distance. Figure 12 shows the paths calculated by the proposed framework. Here, the blue and green UAVs fly a much longer trajectory than the red UAV, but they fly their trajectory at constant altitude whereas the red UAV climbs considerably to visit the two DPs located in the middle of the island. As mentioned in Section 5.1, the vertical speed of the UAV is much slower than its horizontal speed. So overall, the three UAVs have similar flying times. The overall duration of the mission is 788.8 s as listed in Table 3 which is much shorter than that of the previous test. In a third test, paths that minimize energy are calculated. This resulted in paths very similar to those minimizing the flying time.

**Figure 13:**
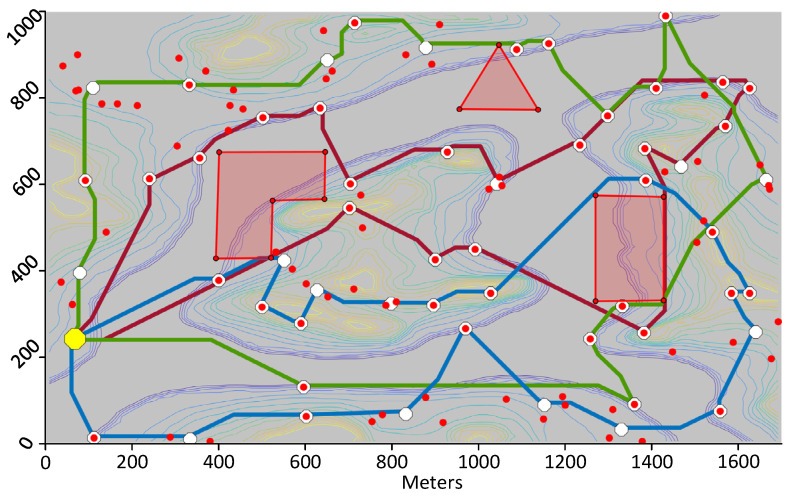
Paths minimizing distance for three UAVs visiting 55 download points covering 100 sensors.

**Figure 14:**
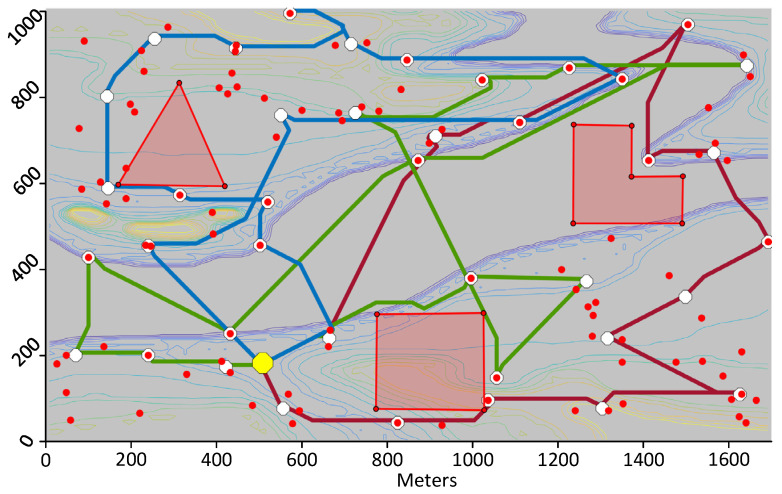
Paths minimizing distance for three UAVs visiting 40 download points covering 100 sensors.

In the third trial, a more complex scenario is shown. The same map is used but 100 sensors are randomly placed with a wireless range of 170 m. The locations of the DPs are then calculated. Initially, the proposed framework calculates 66 DPs, but following the removal of extra DPs at Algorithm 2, 55 DPs remain. The paths for three UAVs that minimize distance are then calculated and plotted in Figure 13. A similar test is run on a different map with again 100 sensors randomly positioned, but this time, the objective function minimized is the flying time of the UAVs. The calculated paths are shown in Figure 14. On that figure, one may see loops such as for the green trajectory, however, the path is in three dimensions and these loops result in shorter flying times.

### 7.2. Test 2: Measuring the Speed-Up of the Parallel Implementation

The second test consists of measuring the speed-up produced by the parallel implementation of the proposed algorithm. First, the focus is on the SSSP algorithm which is the most time-consuming part of the overall framework. For the SSSP algorithm, three implementations to solve the problem shown in Figure 13 are tested. The SSSP is run for every DP and calculates the shortest path to every other DPs. The first implementation tested is the Dijkstra algorithm from the Boost C++ library [36]. This implementation is considered to be highly efficient and is used here as the reference for the sequential implementation. The second implementation is the CUDA implementation as proposed by Harish et al. in [28]. The third implementation is the proposed CUDA SSSP implementation based on Harish et al., but which avoids the atomic operation, which runs the SSSP instances in batch mode to increase the level of parallelism and which run multiple iteration of the while loop at Algorithm 3 before checking the number of nodes in the frontier array.

**Figure 15:**
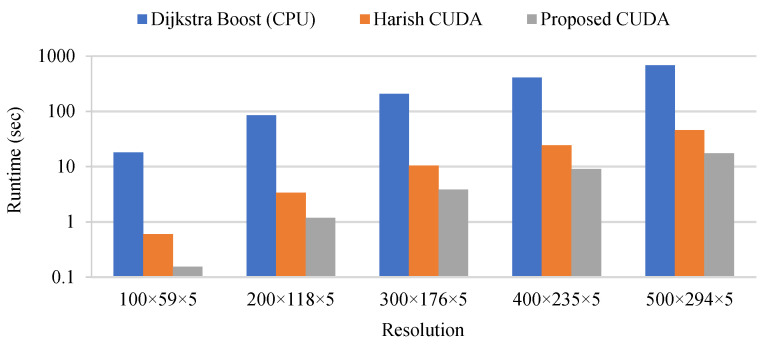
Runtime of the three SSSP implementations tested.

**Figure 16:**
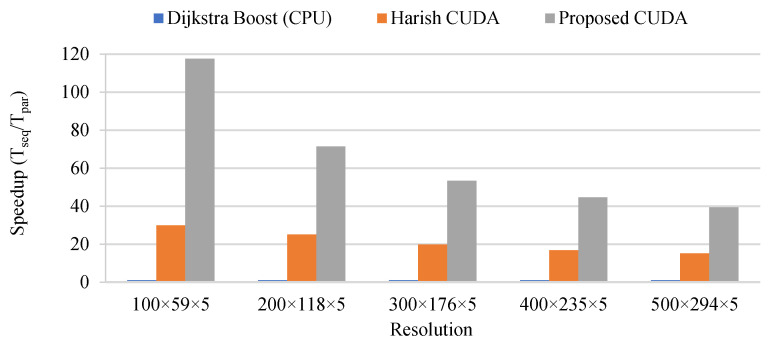
Speed-up of the three SSSP implementations tested.

The three SSSP implementations are tested on a Dell 5820 workstation equipped with a Xeon W-2195 CPU and an RTX-2080 Ti GPU. The CPU has 18 cores and a peak frequency of 3.6 GHz. The specifications for the GPU were given in Section 4. The runtime and speed-up for the SSSP implementations solving 55 instances of the SSSP problem for various map resolutions are shown in Figure 15 and Figure 16. There are 55 instances of the SSSP problem to be solved because the map processed (the one shown in Figure 13) contains 55 DPs. The runtime for the sequential implementation on CPU is 18.0 s for the smallest resolution and 684.9 s for the largest resolution compared to 0.6 s and 45.4 s for Harish’s implementation and 0.15 s and 17.4 s for the proposed CUDA implementation. This translates into speed-ups of 29.9× and 15.1× for Harish’s implementation and 117.6× and 39.4× for the proposed implementation. These speed-ups clearly demonstrate the advantage of the parallel implementation on GPU. However, the reduction of speed-up as the map resolution increases is normal and is caused by the higher complexity of the parallel algorithm compared to the Dijkstra algorithm. By design, the parallel algorithm performs more calculation than the Dijkstra algorithm. This means that if the map resolution would continue to increase, there would be a point where the parallel algorithm and the Dijkstra algorithm would exhibit the same execution time and there would be no advantage for the parallelization. However, for practical map resolutions, there is a significant advantage to the parallel implementation on GPU. From the results obtained, it can also be concluded that the proposed CUDA implementation is much faster than the original parallel implementation by Harish et al. [28]. This is because it avoids the atomic operation, it processes the SSSP instances in batches of 10 and it runs multiple iterations of the while loop in Algorithm 3 before calculating the number of nodes in the frontier array which is an expensive operation.

To assess the contribution of the parallel SSSP algorithm to the overall framework, Table 4 lists the runtime for each function composing the overall framework. It is important to note that the runtime for the parallel SSSP includes the data transfer to and from the GPU. When minimizing for time or energy, filling the cost matrix requires that the path between each pair of DPs be built so that the exact speed and power required can be calculated along the path. This step is parallelized in OpenMP on multi-core CPU using one thread per pairs. For the GA, OpenMP is also used to parallelize each step of the algorithm using one thread per candidate solution. For the other functions, they are intrinsically sequential and are therefore not parallelized. As listed in Table 4, the overall algorithm executes in 20.7 s which represents a 33.3× speed-up compared to the sequential implementation. This is a real advantage when new paths need to be computed in a short time such as when new sensors or obstacles are discovered.

## 8. Discussion

This paper makes three main contributions which also highlight its novelty compared to previous works. The first contribution is to propose a complete framework for the data acquisition from wireless sensors using a team of UAVs. In Section 2 of this paper, a literature survey of recent research works that focused on this problem was provided. Several of the papers focused on one aspect of the problem. Here, the proposed framework covers all the steps from the sensor clustering to the collision avoidance strategy. This last part is critical to any multi-UAV application, but is often omitted in papers related to data acquisition using UAVs.

Several of the papers surveyed in Section 2 defined the problem of wireless sensor data acquisition using UAVs in a 2D environment or used a simplistic representation of the environment. A second contribution made in this paper is that it proposes a solution using a 3D representation with a high resolution of the terrain. The scenarios included a large number of sensors, hilly terrains, and no-fly zones in order to show that the proposed algorithm based on the SSSP and the GA is truly capable of calculating optimized trajectories in difficult and complex 3D environments.

Finally, a third contribution is the parallel implementation of the algorithm on GPU. Given the computational complexity of the proposed framework, its runtime is too long to allow for a quick recalculation when a new sensors or obstacles are detected. To speed up the calculation, a parallel implementation of the SSSP algorithm proposed by Harish et al. [28] was used and adapted to this specific application where multiple SSSP instances needed to be solved simultaneously. This parallel implementation on GPU resulted in a 33.3× acceleration compared to a sequential implementation, a significant advantage when it comes to recalculating the UAV trajectories during the mission.

In their future works, the authors of this paper intend to extend their proposed framework to include a communication network between the UAVs to relay the data collected from the sensors to the ground stations as the data is collected instead of waiting for the UAV to return to the ground station. This will increase the problem complexity as the UAVs will have to remain in communication range between themselves to maintain the network integrity, but the service provided would be far superior. Another research avenue they intend to explore is the implementation of the proposed framework in a field experiment using real UAVs and real wireless sensors. This would raise new challenges such as the imprecision of GPS localization, the limited computation capability of the UAVs, the limited bandwidth of the UAV-to-UAV communication, the management of hardware faults and the scheduling of UAV battery recharging. Moreover, in this present paper, the position of the sensors was assumed to be initially known. In a real scenario, the sensors would have to be discovered by the UAVs who would initially fly a search pattern over the terrain. All these considerations add complexity to the problem of data acquisition from wireless sensors using a team of UAVs when it comes to a practical implementation. However, the proposed framework is a step forward towards a possible solution.

## 9. Conclusions

This paper presented a framework for the path planning of a team of UAVs tasked to download data from wireless sensors scattered over a 3D environment. The framework uses a multi-step approach which relies on an iterative k-means algorithm to group the sensors into clusters whose centroids represent the DPs, locations where the UAVs will position themselves to download the data from the sensors. The framework then builds a sparse graph and uses an SSSP algorithm to find the optimal path connecting each pair of DPs. Following that, a GA is used to assign the DPs to the UAVs and to order the DPs to form a tour for each UAV. A collision avoidance strategy which relies on adding delays at the DPs is implemented. The final result is a collision-free path for each UAV that visits all the DPs in an optimized manner. Some of the steps of the proposed framework including the SSSP algorithm are parallelized on GPU using CUDA and on CPU using OpenMP and allows for the calculation of the paths in 20.7 s, a 33.3× speed-up compared to a sequential execution on CPU. To summarize, this paper makes three main contributions. Firstly, it proposes a complete framework for the data acquisition from wireless sensors using multiple UAVs. Secondly, compared to previous works, it expands the solution from 2D to 3D environments. Thirdly, it proposes a parallelization strategy to parallelize the algorithm on GPU using CUDA and on multicore CPU using OpenMP.

## Figures and Tables

**Table 1:** Example of a Cost Matrix.

	To	1	2	3	…	*N*
From						
1			C_1, 2_	C_1, 3_		C_1, *N*_
2		C_2, 1_		C_2, 3_		C_2, *N*_
3		C_3, 1_	C_3, 2_			C_3, *N*_
…						
*N*		C_*N*, 1_	C_*N*, 2_	C_*N*, 3_		

**Table 2:** Statistics for the Paths Shown in Figure 10.

UAV	Distance (m)	Time (s)	Avg Speed (m/s)	Energy (kJ)
Blue	2548.2	652.9	3.9	64.232
Red	4406.4	899.5	4.9	126.806
Green	2811.3	729.3	3.9	95.674

**Table 3:** Statistics for the Paths Shown in Figure 12.

UAV	Distance (m)	Time (s)	Avg Speed (m/s)	Energy (kJ)
Blue	4449.0	788.8	5.6	101.651
Red	2252.4	686.2	3.3	84.833
Green	4096.6	774.5	5.3	94.521

**Table 4:** Runtime and Speed-up for the Proposed Wireless Sensor Data Acquisition Algorithm (3 UAVs, 55POIs, and resolution of 500 × 294 × 5).

Operation	Runtime (s)		Speed-Up
	Sequential	Parallel	
Positioning the DPs	0.097	-	1.0×
Build CSC graph	0.447	-	1.0×
Reduce graph	0.223	-	1.0×
Build CSR graph	1.847	-	1.0×
SSSP	684.898	17.612	38.9×
Fill cost matrix	0.314	0.050	6.3×
Genetic algorithm	2.424	0.375	6.5×
Build final path	0.049	-	1.0×
Collision avoidance	0.029	-	1.0×
Total	690.328	20.729	33.3×

## Data Availability

Not applicable.

## Nomenclature

*tid*	thread identifier
*n*	number of nodes
*m*	number of edges
***s***	index of *start* node
*e*	index of *end* node
*sum*	sum of the values in *F*
*G*	graph in sparse CSR format
*W*	edge weight
*F*	flag array of size *n*
*C*	node cost array of size *n*
*U*	node updating cost array of size *n*
